# Mapping Institutional Interventions to Mitigate Suicides: A Study of Causes and Prevention

**DOI:** 10.3390/ijerph182010880

**Published:** 2021-10-16

**Authors:** Zia Ullah, Nighat Akbar Shah, Sonia Shamroz Khan, Naveed Ahmad, Miklas Scholz

**Affiliations:** 1 Faculty of Business Administration, Lahore Leads University, Lahore 54000, Pakistan; 2 Adur Productions LLC, Memphis, TN 38125, USA; contact@adurproductions.com; 3 Chitral Police Service of Pakistan, Chitral 17200, Pakistan; sonia.shamroz@gmail.com; 4 Faculty of Management Studies, University of Central Punjab, Lahore 54000, Pakistan; naveeddgk2010@gmail.com or; 5 Department of Management Sciences, Virtual University of Pakistan, Lahore 54000, Pakistan; 6 Division of Water Resources Engineering, Department of Building and Environmental Technology, Faculty of Engineering, Lund University, 221 00 Lund, Sweden; 7 Department of Civil Engineering Science, School of Civil Engineering and the Built Environment, University of Johannesburg, Kingsway Campus, Auckland Park 2006, Johannesburg 2092, South Africa; 8 Department of Town Planning, Engineering Networks and Systems, South Ural State University (National Research University), 454080 Chelyabinsk, Russia; 9 Institute of Environmental Engineering, Wroclaw University of Environmental and Life Sciences, 50-375 Wrocław, Poland

**Keywords:** suicide, violence, mental health, family structure, complaint center, litigation

## Abstract

Suicide is an extreme, tragic act and an important subject for social inquiry. It is the rising public health issue prevalent in the Himalayan range of Pakistan. The young and educated population is more prone to suicide instead of using this prime phase of age productively. Unfortunately, the suicide problem remains unaddressed, the causes remain undefined, solutions are not in the works, and in situations when others play a part in driving someone to commit suicide, no one is being held accountable. This study is aimed at uncovering the root causes of suicide and proposing some preventive measures to mitigate the problem. Our team studied three years’ worth of data (2017–2019) on suicides from the office of Human Rights Commission of Pakistan, Chitral. In addition, we conducted semi-structured interviews of different stakeholders, including family members, neighbors, lawyers, and police personnel. The findings revealed that extended family pressures, the power dynamic between sustainers and dependents, family conflicts, and inheritance cases were the major causes of the domestic violence that preceded suicide attempts. Mental health issues, forced marriages, academic competitions, and flawed litigation processes were also among the leading causes of suicide. Awareness through education and religious sermons, strengthening healthcare organizations, restructuring family systems, establishing police complaint centers, effective prosecution processes, and imparting lifesaving skills have been identified as measures to prevent suicide. This study has theoretical and practical implications, as it adds certain novel variables regarding the causes and solutions of suicide to the existing body of literature and guides public authorities to strengthen institutions to intervene effectively.

## 1. Introduction

No life is free of problems, and during each individual’s life cycle, the volume and intensity of problems vary. However, a physical, cognitive, affective, and social configuration of personality causes every individual to tackle problems differently [1]. Some people manage their problems proactively, while others are reactive. Some people attempt to overcome problems, while others try to escape them. Some people transform their problems into opportunities by studying the causes of the problems scientifically, planning properly, thinking positively, and finding and evaluating alternative solutions [2], while other people under problematic conditions lose their cognitive efficiency and emotional stability, become blind to alternate solutions, and, eventually, become completely overwhelmed [3]. Those who become overwhelmed consider suicide as their only solution. Despite the consistent developments in neurosciences, along with an increase in the understanding of human behavior pathophysiology, suicide remains a perplexing challenge [4].

Creating a situation that results in the suicide of another is considered to be a criminal activity, and the state will prosecute any individual charged with this offense as per the law of the land [5,6]. However, suicide cases are rarely tried in the courts. Suicide events are seldom thoroughly investigated to ascertain the causes, and people involved in driving the victim to suicide are not generally identified or held liable. If an investigation that could lead to legal proceedings is initiated, immediate family members and other relatives usually prefer to terminate the related litigation. A suicide event is considered to be a matter of dishonor and humiliation for the entire family and clan [7,8]. Consequently, family members strive to diminish or erase the suicide event as quickly as possible [9]. Law enforcement agencies, including police and the judiciary, have a pattern of concluding those cases without an in-depth inquiry and investigation of the causes and possible perpetrators. The claim that the deceased person was insane or facing domestic violence or social incongruence is considered sufficient justification to dispose of the legal proceedings.

As suicide cases are not scientific studies, solutions to control suicide have not yet been placed. The context at hand invites research attention to carefully study the growing problem of suicide. Suicide is a social issue, and its causes are profoundly embedded in the social structure. Since social structures, including culture, education, and lifestyle, are not identical among nations, the causes of suicide may vary from society to society. For example, a leading cause of suicide in Europe is the use of alcohol [10,11], whereas this is not the case in other cultures, as drinking alcohol is legally banned in Pakistan. In the same way, poor social integration has been identified as another significant reason for suicide in Europe [11,12], but this is not a prominent cause of suicide in Pakistan. In Europe, the suicide rate of males is higher than that of females [13,14]; however, in other cultures, females are more prone to suicides. Thus, it is quite unreasonable to generalize the findings on suicide for different social structures. Therefore, these kinds of studies are vital to understanding the causes of the rising suicide problem in Pakistan’s northern region.

In the face of an alarmingly increasing trend of suicides in the Chitral District of Pakistan where crime rates are relatively minimal, the present study aims at analyzing the suicide cases that occurred during the last three years (2017–2019) and uncovering the factual causes. This study discusses the role of different institutions and their interventions to address the problem. This study identifies some key causes and proposes some remedial measures to address them. This study highlights gaps in the investigation and prosecution, as well as some social pressures that do not allow the authorities to take suicide cases to their logical conclusions. To arrive at these conclusions, we collected three years’ worth of data on suicides from the Office of the Human Rights Commission of Pakistan, Chitral, ranging from 2017 to 2019. We did not find reliable data on suicides before and after this period. Data on suicides for the period before 2017 were not properly documented and data for 2020 had not yet been released by the office. We interviewed family members of suicide victims (two husbands, a mother, a father, and a brother); two lawyers who had been engaged in litigation of five suicide cases; two chief physicians who know the treatment aspects of patients with suicide ideations; two police personnel who had experience investigating more than ten suicide cases each; and five members of the society (from the neighbors of suicide victims) to gain insight and more detailed information regarding suicide.

Suicide is a widespread phenomenon. More than one million people lose their lives annually, and three-quarters of suicide victims belong to low- and middle-income countries (LMICs) [15]. Suicide is now the second-leading cause of death among adolescents and youths (15–29 years), which are considered to be the most productive years in the lifespan of an individual [16,17,18].

In Muslim countries, suicidal deaths are reported as “Other Violent Death (OVD)”, because suicides are religiously and culturally condemned. As a result of this condemnation, low suicidal rates are reported; however, the exact data do not exist [19,20,21]. The estimated age-standardized suicide rate in Pakistan is 4.4 per 100,000 people [22]. The suicide death rates in neighboring India, Bangladesh, and Sri Lanka are 13.33, 5.73, and 7.55 per 100,000 people, respectively [23]. Despite the low estimated rate, recent data suggest that suicide is becoming a significant public health problem in Pakistan [24].

Suicide is a complex phenomenon that occurs from a multidimensional interaction of genetic, biological, psychological, and environmental factors. The majority of suicides are said to have occurred because of poverty and economic suffering [25,26,27]. Khan [24] identified religious and prevailing legal factors as reasons for attempted and completed suicides in Pakistan. However, in the majority of suicidal cases, it is difficult to obtain accurate data regarding the presence of the prevailing social, religious, and legal factors [28]. There has been increasing concern over the relative importance of social factors in suicide, such as poverty and gender discrimination that includes humiliating behavior towards females. In many countries, these factors are the leading causes of attempted and completed or successful suicides [27,29].

Critelli and McPherson [30] identified marital incongruence and domestic violence that generally results in separation and divorce as the causes of attempted or committed suicide. In the same way, the most commonly cited traumatic events of young females in India were unsatisfactory marital lives, economic problems, and violence by spouses [31]. Thus, women living under domestic and gender violence were more inclined toward suicide [32]. Furthermore, women living in patriarchal societies like Afghanistan, Pakistan, India, and Bangladesh are more vulnerable to completing or attempting suicide [33,34]. Most research states that interpersonal relationship disputes, domestic conflicts, and economic problems are the fundamental reasons for suicides in Pakistan [19,35,36]. However, Khan [24] highlighted depression as the prime cause of attempted and completed suicide. Additionally, conjugal relations also appear to be a major source of stress (especially for females), resulting in high psychological morbidity and suicidal attempts [37].

Rahnuma, Fangtong [38] claimed that rapid social change brought about a marked shift in Northern Pakistani culture and society, which transformed the community from pastoral to agricultural life and, then, to government and corporate employment, further evolving towards a merchant society, followed by a capitalist society. This transformation resulted in a shift of societal values from collectivism to individualism. Individuals are left alone to cope with their problems, with decreasing social support. This isolation often fuels frustration and alienation, which are the main risk factors for suicide [39].

As unemployment is strongly correlated with suicide, unemployed people, particularly males, are more prone to self-harming behavior and suicidal intentions in Pakistan [40]. Thus, joblessness and the prevailing bleak conditions and growing poverty carry significant policy implications as macro-level risk factors for suicide [41,42]. Likewise, it has also been reported by Sheikh [43] that low academic achievement is significantly associated with suicide. It is believed that low academic attainment intensely influences an individual by adversely affecting their ability to cope with stress, compete in the job market, and improve their social status [44]. Further, Ahmed, Bhati [19] reported the reasons for suicide in Ghizer Valley (North Pakistan), such as social factors (e.g., educational pressure, economic constraints, divorce, and interpersonal problems); cultural factors (e.g., lack of decision-making power, inadequate personal freedom, and demand for a male child); and psychological (mental illness and depression). Few research studies reported that academic pressure, unrealistic expectations of parents, and hopelessness were the central reasons for suicide in the northern areas of Pakistan, including Ghizer [45,46] and Hunza [47]. As a result of increased educational services, the introduction of modern technology, and improvement in the overall literacy rate in the northern belt of Pakistan, the existing social structure of the area suffers from the conflict between traditional social structure and modernity [48]. This conflict became apparent in researching suicidal issues in Northern Pakistan. Only one study reported domestic violence as the main factor for female suicide in this belt [46].

Unaffordable tuition and stiff competition among students due to the rigorous atmosphere of educational institutions, combined with an insufficient social support system, have resulted in higher-level stress amongst students [28,49]. Therefore, the fear of failure, peer pressure, and teachers’ and parents’ admonitions are the key factors that underlie suicidal behaviors of students in Pakistan [50]. Another reason is cultural and socioeconomic disparity, as students in educational institutes come from a wide spectrum of socioeconomic and cultural backgrounds [28]. These disparities make students status conscious and create a sense of competition among them, adding to the stress of academic competition [51].

Studies of the given population showed that Kalash, the indigenous community of Chitral, Pakistan, did not back the notion of suicidal death, as that culture traditionally believes that life is a gift from God, trusted to them to do good deeds, and they have no right to destroy it [52,53]. Suicide cases did not exist in that area until 1988 [19]. However, after 1996, suicide cases became common and relatively frequent. Usually, completed or attempted suicidal cases are associated with the event of a failed romance, which can cause a fatal level of anxiety and depression [47,48], and even an occasional heart attack [54]. It has also been reported that honor killing cases in Chitral were reported as suicide cases in order to help the perpetrators escape legal punishment [55]. Our findings showed that, in some cases, the parents of married women who committed suicide sued the husbands of the deceased women for murdering their wives and reporting their deaths as suicides [56]. Husbands have been caught killing their wives for suspected infidelity, disloyalty, or attempting to prevent the husband from pursuing another marriage [55]. Additionally, it has been documented that the arranged marriages of Chitrali females with Punjabi males and the subsequent mismatch between the couples has resulted in suicidal attempts [57]. Apart from these incidents, in Pakistani society marriages against the parents’ wishes are assumed to be a matter of disobedience, and elopement and court marriages are often looked upon as a great sin [55,58]. Under such circumstances, the marrying couples are socially isolated and pressured in a manner that can drive them to suicide [59].

### Research Framework

The central theme of this research is to investigate the phenomenon of suicide in detail. The literature presents causes of suicide that are superficial and greatly oversimplified to the point that effective corrective actions cannot be devised based on these current findings. Thus, we found it imperative to come up with answers to the following questions apart from demographic analysis of the suicide victims:

1. What are the actual causes of the rising trend of suicide, and suicidal attempts, in both districts of Chitral?

One of the major causes of suicide is domestic violence [60,61]. Further questions arise as to the underlying causes of domestic violence and those who behave violently. Second, mental health and mental retardation are identified as common reasons for suicide [62]. Again, the question arises as to the underlying causes of mental health problems that make a person suicidal. Third, financial and economic factors are held liable for suicide [63,64]. This leaves the question as to why poverty is allowed to be severe enough to drive people to take their own lives. Lastly, failures to achieve predetermined goals, including failure to meet academic targets [28], failure to obtain dream jobs [44], and broken love affairs [48], are generally identified as the causes of suicide. Hence, the need to understand the social pressures associated with these targets is also addressed in this study.

2 Why do perpetrators who cause others to resort to suicide go unpunished, and why is no one is held responsible for this crime?

Suicide is an extreme action, and certainly, there are many invisible hands behind completed and attempted suicides. Conditions are created under which survival seems impossible; hence, suicide becomes the solution. People who are deliberately involved in creating such fatal conditions unfortunately are not held liable. Thus, the absence of fear of punishment encourages certain people to create a highly stressful environment where suicide seems like the only possible escape for the victim. The unanswered question is why are these criminals not subjected to prosecution and punishment.

3 What are the roles and degrees of interventions of the concerned institutions?

Regardless of the specific motivations for suicide, the person perpetrating suicide reaches the peak of depression and anxiety at that fatal moment. Depression plays a pivotal role between any cause(s) of suicide and the suicidal act itself. The question thus arises as to why psychological and psychiatric interventions are not available for emotional and mental therapy? Typically, investigating agencies—particularly, the police—do not investigate suicide cases with the intention of determining the actual reasons. Courts dispose of such cases due to insufficient evidence and witnesses. The role of educational and religious institutions is also insignificant in creating awareness and preventing suicide. We want to understand why these institutions are dormant in this regard and how it will be possible to activate them to play their part to combat this growing social problem.

## 2. Methodology

Chitral Valley is situated in the extreme northwest of Pakistan and has 500,000 inhabitants. It is considered to be the most peaceful part of the country, and the crime rate is comparatively much lower there than in other regions of Pakistan. The literacy rate of both genders is high, and females have a relatively higher representation in the workplace. Despite these positive indicators, the suicide rate is considerable and trending upward. This alarming issue receives mere condemnation, and no serious remedial steps from any quarter have been observed to date. Keeping in view all of these facts, Chitral is being used as the population for this study.

Our team used both primary and secondary data to answer the research questions. The last three years’ (2017–2019) reports on suicide were retrieved from the Office of the Human Rights Commission of Pakistan, Chitral. The data carried 49 committed suicide cases, their demographic details, causes of suicide, and methods of suicide. The data for this period were officially verified, and the data for 2020 were yet to be released. This data were analyzed using cross-tabulation through SPSS. The data were divided into many tables according to demographic distribution, reported causes, and mode of suicides.

In addition to this data, we conducted semi-structured interviews of 16 respondents after obtaining informed consent. The respondents were selected through the purposive sampling method. The respondents consisted of five family members of different suicide victims, two police personnel with experience in investigating suicide cases, and two lawyers. Additionally, two clinicians were interviewed. The matter was also discussed with community members (five respondents) to obtain independent views. In-depth interviews were conducted using English, Urdu, and the local language, as per the respondents’ convenience. The responses were recorded, and the duration of the interviews ranged from 25 to 35 minutes. Ethical standards were followed while collecting data. Informed consent was obtained from each respondent to participate in the survey willingly. In addition to this, approval from the ethical review committee of Lahore Leads University, Pakistan, was obtained under letter No. LLU/ERC/Res/21/28 on 29 April 2021 to conduct the proposed survey.

After conducting all of the detailed interviews, the authors transcribed the data and made a thematic analysis to identify patterns within the responses. We extracted certain root causes of suicide that were not yet identified by the researchers (interview themes are in Table 1).

## 3. Results

### 3.1. Interviews

Data collection was made from sources, interviews, and reports on suicides prepared by the Human Rights Commission of Pakistan Chitral Office. We aimed to reach actual and root causes of suicide instead of relying on the reported apparent causes. Therefore, we conducted semi-structured interviews. The respondents discussed various aspects and issues regarding suicides. Family members of the suicide victims told about the actual root causes of domestic violence and marital issues and suggested certain remedies. Clinicians highlighted mental ailments that cause suicide and suggested possible solutions to overcome these kinds of issues. Lawyers and police personnel identified the lacunas that exist in the investigation and litigation system. The society members highlighted economic factors, academic factors, and domestic violence. However, the responses mostly complemented or overlapped one another (Table 1).

### 3.2. Quantitative Data

The quantitative data were analyzed using cross-tabulation. We bifurcated the data into males and females and analyzed it against the demographic characteristics and reasons for suicides. It was found that suicide is on an increasing trend, and the figures have more than doubled in the three-year period. Females are more inclined toward suicide, and during the given three years, 60% were female. In the same way, the trend of suicide is increasing in females year by year (Table 2).

Table 3 shows that suicide is more prevalent in early ages. Fifty-seven percent of suicides take place during 14–26 years of age. This is usually considered to be the prime and most productive period of life. Females at an early age are more inclined toward suicide compared to males. As age increases, the inclination toward suicide in females decreases, but the male trend in suicide is equally dispersed in all age groups.

The majority of those who have carried out suicide (66%) are educated. However, their education level averages at less than or equal to 10 years of education. As their education goes beyond 10 grades, the ratio of suicide radically decreases. Half of the female cases were illiterate, and in the same manner, as their education increases, the suicide trend in females also decreases (Table 4).

In terms of occupation, jobless males are more prone to suicide, and more than half of the total males who died were unemployed. The majority of females who died from suicide were housewives. A considerable ratio (20%) was students (Table 5). However, the trend toward suicide in job holders and farmers is low.

Table 6 shows the marital status of those who committed suicide. The data show that the majority (67%) were unmarried. Both unmarried males and females completed or attempted suicide more than those who were married.

The most prevalent suicide method was drowning in rivers, and the majority of males and females adopted this method (Table 7). In some cases, the dead bodies could not be recovered. The second-most frequent method of suicide by men was by a firearm, whereas females used poison to end their lives. Drowning, firearms, and poison are thought to be quick and fatal, which is why they are chosen most often for suicidal purposes. Only two males and one female hung themselves. These methods are quick and fatal, which is why these methods are mostly preferred for suicide.

Three major reasons for suicide are reported in the data (Table 8). The major reason is mental illness and retardation. The majority (55%) of the deaths were caused by mental disorders, including depression and insanity. Mental illness is cited as the major cause for both genders. Domestic violence is the second major cause (25%) for both genders. Four suicides were caused by a failure in academic examinations or a failure to secure expected grades in examinations, particularly at the time of matriculation and intermediate-level examinations. The reasons for six suicide cases could not be determined and are shown as “unknown” on official documents.

Our results identified the extended family system, mental health issues, economic disparity, a flawed litigation system, forced marriages, and academic competition as the leading causes of suicide. Awareness through education and sermons, police complaint centers, the proper prosecution process of suicide cases to convict perpetrators, restructuring extended families into small unit families, and imparting lifesaving skills were highlighted as preventive measures to minimize suicides.

## 4. Discussion

The context and population we studied is relatively peaceful, with records that show the least crime occurrence [8]. Despite the high literacy rate, this population is experiencing an increasing trend in actual and attempted suicides [9]. Young and educated people commit suicide, yet no preventive measures have been established. Many organizations, including law enforcement organizations, judiciary, human rights activists, NGOs, and clergy, are supposed to alleviate this social issue. However, their contributions in this regard are insubstantial and do not play a significant role to address and mitigate the problem. The actual causes of increasing suicides are still obscure, and reasons such as domestic violence and mental retardation are superficially attributed to suicide without deeper examination. Our findings showed that the majority of the parties, including prosecuting agencies, declare the deceased as mentally insane to avoid the criminal trial process.

The literature does not present the root causes of suicides in the northern region of Pakistan. A few generic reasons such as domestic violence [34,48,65], mental diseases [66,67,68], economic reasons [63,64], competition in academic fields [28], and broken love affairs [48] are usually coined as the reasons for suicides. Understanding these generic causes does not allow us to devise effective solutions. On the other hand, our study takes a microscopic view of the root causes of marital discord, financial circumstances, and other issues that lead to suicide. Based on this analysis, this study also proposes solutions to mitigate the frequency of suicide.

Causes of suicide can vary from place to place, from time to time, and even from person to person. Therefore, these causes need to be studied in relation to the context and other contributing factors in order to identify and correct them. It is quite unfortunate that, in this given area, the reasons for suicide have not been properly studied and documented. The reasons mentioned in certain documents are superficial, oversimplified, and appear to have been presented to minimally satisfy certain legal requirements.

A major cause of suicide is domestic violence [34]. However, domestic violence has many dimensions and shapes, which the previous studies did not uncover. Our study, mainly based on interviews, unearthed the following root causes that contribute to domestic violence:

- The joint family system was found to be one of the major causes of domestic violence. A joint family is a family where more than one couple and their children live. Joint families become relatively large in size, and problems arise pertaining to the division of work, role ambiguity, and allocation and possession of resources. The tug of war for authority and resources creates conflict among family members. The perceived inappropriate family structure and hierarchy cause deviance and conflict that lead to acute domestic violence. Most victims of domestic violence are female.
- The conflict between the sustainer and the sustained usually causes violence. In most cases, it is found that a handful of the family members earn and provide sustenance to the family, but the majority of the family members do not engage in any economic activity. These few individuals carry the entire economic burden of family. These earners work for hours and put in greater exertion and effort to earn a living; however, the beneficiaries or dependents lead comfortable, often luxurious, lives at the expense of those who earn. Economic inequality causes conflicts that, in many cases, eventually lead to domestic violence.
- The inheritance and heredity of paternal and maternal properties also generate violence. In the context at hand, females are not usually given their due portion in inherited properties. Most of the women do not raise a voice against this injustice. Those who claim their due portion in the legacy are commonly confronted and denied. This imbalanced situation can foster violence and bitter consequences.
- Our research has shown that the relationship between mother-in-law and daughter-in-law is not commonly amicable. We found that some mothers-in-law have intolerant attitudes and display aggressive and discourteous behavior toward their daughters-in-law. This inconsistent and incongruent relationship contributes to burnout, depression, and in many cases, domestic violence.

Mental health issues, including chronic mental diseases, depression, anxiety, and fear, were reported to be a major reason, and most of the cases we studied were due to mental health problems. Every person committing suicide will, of course, be in a mentally disheartened state at the time of suicide due to overwhelming and devastating depression levels, irrespective of the root cause. Therefore, we believe that depression itself cannot be blamed as the overall cause of suicide. The root causes of suicide are the underlying factors that cause the fatal depression, not the depression itself. We define mental health issues as the mental disorder or mental ailments that cause deviant, uncivil, and criminal behavior, aside from suicide. Thus, to some extent, the authors disagree with the data report as mental health being the major reason for suicide. On the one hand, it is observed that people with mental disorders are commonly found in the streets, and though they do behave abnormally, they do not usually commit suicide. Mentally retarded people may not be educated, may not be employed, and may not get married. Therefore, these indicators show that majority of the suicide victims were mentally sound and behaving normally. However, some people suffering from permanent and incurable diseases have killed themselves to escape the pain and suffering. In any case, declaring a suicide victim mentally retarded is an easy way to satisfy the court and other stakeholders.

Marital complications were also highlighted as the cause of suicide. Marital-related decisions of girls are mostly made by their guardians. Sometimes, this happens to males as well. Undesirable marriages have lifelong consequences, and both parties suffer from emotional dissonance. Another matrimonial issue comes into play when females are married outside the district. Marriages in Chitral are not bound by the red tape of socioeconomic and cultural formalities. Consequently, people from other parts of the country come to Chitral to obtain wives with relative ease. There is no sound mechanism to properly investigate the socioeconomic status of the suitors, and they sometimes do not provide correct information regarding their socioeconomic standings. Many women who experience these ill-advised marriages lose their desire to live and commit suicide. Another reason for suicide is the advice and direction of parents, particularly the father, to remain married at all costs, no matter how unfavorable the conditions are. Divorced females are not welcomed in their parental homes. Unsuccessful love affairs also cause suicides. When a love affair concludes without marriage, it creates frustration, hopelessness, and depression. Thus, these marital conditions cause acute depression and lead to suicidal ideation and attempts.

Competition in academic performance has also been a cause of suicide. Students seeking admission to medical colleges, engineering colleges, or other highly reputed educational institutions face stiff competition due to limited seats. Parents pressure their children to become medical doctors or engineers to have a respectable and prosperous life and caution them that if they make other choices, their futures will be meaningless. This pressure often becomes so strong that failing to secure admission to the desired educational institution results in suicidal ideation and attempts.

Economic factors have also been identified as reasons for suicide. Our respondents told that the main reason was the inability to repay loans. Joblessness was given as another reason for attempting suicide. However, we did not find any suicide cases due to lack of basic necessities or starvation.

### 4.1. Proposed Remedial Measures

Based on the findings of the study, we extend our recommendations to address the issue of suicide:

#### 4.1.1. Awareness and Education

Educational institutions can play an effective role in addressing the issue of suicide. Suicide is often due to ignorance of the importance of life and of solutions to the problems that drive people to suicide. In our educational institutions, lectures should be delivered on the importance of life and the expected role of each individual in the family, community, and society at large. Teachers should instill the philosophy that life is precious and cannot be destroyed. Death is inevitable and will come, but nature has not left that moment to the discretion of the individual. Second, it must be emphasized that every problem has a solution, and every difficult situation has a way out. People must be empowered to search for solutions rather than escape by suicide. In addition to imparting these ideas, educational institutions can provide counseling and advisory services to the students. These services can help students identify personal aptitudes and potentials for career planning and show them other career choices that can lead to prosperous and respectable lives beyond medicine and engineering. This kind of service will neutralize the stress and depression caused by intense academic competition. Such education and awareness are currently not provided by educational institutions in the manner described above.

#### 4.1.2. Health Institutions

Mental health disorders are the major cause of suicide. Hospitals and other health organizations should provide clinical, psychiatric, and psychological therapies to those with suicidal ideation. The hospitals in this region currently have no psychiatrists and psychologists to treat depressed patients who ultimately commit suicide. Hospitals should provide mentally ill people medical treatment, whereas psychiatrists and psychologists should treat depressed and emotionally disordered patients.

#### 4.1.3. Management of Family Structure

Joint families should be configured into small units, preferably a family consisting of a couple and its children. It is observed that, in small families, parents take complete ownership and responsibility for the family’s care and do not indulge in any conflict or manipulative behavior. Small families were found to be more satisfied irrespective of their socioeconomic conditions. Thus, one of the effective ways to tackle domestic conflicts and violence is by abandoning joint families and adopting and promoting small unit families.

#### 4.1.4. Use of Religious Centers

Clergy can play a vital role in managing the widespread issue of suicide. The population of the area is keenly inclined toward its belief systems and religions. The majority of the population is Muslim, and Islam condemns suicide and declares it to be an egregious sin. Religious leaders should include this topic in their sermons and highlight the devastating consequences of suicide and discuss possible ways to exit bad situations and overcome harsh conditions. In this given context, the role of mosques and Jamat Khana would be effective in tackling the growing suicidal trend.

#### 4.1.5. Police Complaint Centers

The establishment of a police complaint center where people facing violence, including domestic violence, blackmail, deceptions, frauds, and threats, can report such crimes. This center would ensure the safety of the victim and deal with culprits to the fullest extent of the law. Providing these protections would prevent suicides while offering a twofold purpose: protecting the victims and punishing culprits while also providing counseling to victims that would help them restore their lives.

#### 4.1.6. Trial of Suicide Cases

This study revealed that suicide cases are not properly prosecuted in the court of law. Suicide cases are perceived to be a matter of humiliation and disgrace to the family and relatives, so the family members endeavor to dispose of the case as quickly and quietly as possible. Consequently, suicide cases are not pursued or investigated with the true intention of determining, convicting, and punishing the culprits. We propose that, under law, the state would become a party in suicide cases, and the state would be represented by the police and public prosecutor. The state representatives in the court of law would have an obligation to pursue the case even if the family member and relatives do not follow the case actively. Suicide cases need to be investigated and prosecuted more aggressively, and both the family and state should play their due roles in combatting this social problem.

#### 4.1.7. Economic Salvation

Microfinance banks and departments of Zakat are the financial institutions working for the alleviation of poverty. These organizations can work more effectively by collecting information about the people in desperate economic situations and helping them at the right time. Promoting an entrepreneurial mindset and providing the necessary equipment for entrepreneurship will produce lasting positive economic outcomes. This can be done in collaboration with business schools and financial institutions, including private banks.

#### 4.1.8. Lifesaving Skills

Interviewing survivors of failed suicide attempts to develop a better understanding of the suicide victim’s mindset and the process he/she went through to take this desperate step. Since drowning is a common suicide method, teaching people to swim at a young age could give those who made an impulsive decision the chance to survive even after the initial act. Educational institutions should also teach the student what to do after taking poison and other survival tactics related to suicide attempts. As an immediate response in this regard, educational institutions can include swimming in their sports activities.

## 5. Conclusions

Suicide is alarmingly increasing in the northern part of Pakistan, particularly in the Valley of Chitral. Young, educated females are more prone to suicide. Unfortunately, suicide cases are neither studied scientifically to determine the factual causes nor are the cases properly prosecuted in the court of law. This study aimed at unearthing the actual causes of suicide and devising preventive measures. Based on the secondary data on suicide obtained from the office of the Human Rights Commission of Pakistan, Chitral, and interviews, the study revealed certain compelling findings. We found that the reported causes of suicide were oversimplified and superficial. Joint family systems, inheritance, heredity, economic disparity within the family, and mother-in-law versus daughter-in-law conflict were the source of domestic violence that led quite a large number of people to attempt suicide. Mental health issues, including depression, anxiety, and other mental ailments, also create suicide ideation and suicide attempts. Forced marriages, the collapse of love affairs, and failures in academic competitions were the major reasons for the increasing suicide in the given population.

Based on our findings, we suggest some remedial measures. The academic curricula should include lessons on suicide. Everyone should know that suicide is not the solution to life’s problems, there are better alternatives, and one should search for them before resorting to suicide. One should realize that life is too valuable to be destroyed to escape an unfavorable situation. Health and religious organizations can effectively intervene to address the issue. Hospitals should employ neurologists, psychiatrists, and psychologists for medical and emotional therapy. Religious leaders should also condemn suicide and suicidal ideations through their sermons. The establishment of police complaint centers would serve as a proactive measurement to deter the problem. Proper and complete litigation to convict the culprits would serve as an example for others. Lifesaving training including swimming should be publicly provided; this would aid those who immediately regret an in-process suicide attempt.

## 6. Implications

The increasing tendency of suicide in the selected population is alarming, and it becomes more dangerous when the young and educated strata of the society become prone to it. In the face of such distressing circumstances, the findings bear implications for researchers, government agencies, educators, healthcare providers, and the members of society at large.

- The findings of the study add some new variables to the existing literature. These variables are the factual reasons for suicide in the given population. These variables include causes of domestic violence, marital issues, health issues, and economic issues, which are invisible as such.
- The findings add certain facts to the literature, including the demographic composition of those who completed suicide, the largely prevalent causes of suicide, and proposed remedial measures to prevent and combat the issue of suicide.
- The study guides the concerned public authorities to strengthen the healthcare organizations by placing neurologists, psychiatrists, and psychologists for medical intervention to prevent suicide. There are two public sector district headquarter hospitals with positions to hire neurologists, psychiatrists, and psychologists; however, these vacancies are lying vacant. The provincial health department is responsible for filling these positions.
- The findings also urge public authorities to incorporate lessons regarding the issue of suicide into the curricula of educational institutions. National and provincial curriculum development and review committees can include teachings on the disadvantages of suicide and possible ways to avoid suicides.
- The study exhorts religious leaders to denounce the act of suicide as a social problem and encourage people to live a delightful life, as nature provides countless alternative ways of life. The population of the area consists of two major religious sects, Mosques and Jamat Khana being their worship places, where clergies deliver their weekly sermons. People of both sects are quite religious and follow the instructions of the clergies. So, the inclusion of lessons regarding suicide in the Friday sermons will definitely influence society.
- The study recommends that law enforcement agencies establish a platform where oppressed, tyrannized, maltreated, and deceived people can report their complaints; timely addressing the complaints will help prevent suicidal acts. We propose a complaint cell under the patronage of police. The cell can provide counseling and guidance on how to solve problems and obtain justice.
- The study suggests that the court of law make the litigation process more effective in convicting perpetrators. Merely disposing of cases based on insufficient evidence encourages perpetrators to repeat their tyrannies. In every district court public prosecutors represent the state and plead cases of criminal natures. So public prosecutors should take the responsibility to litigate suicide cases properly with an aim to reduce the occurrence of suicide.
- The study suggests concerned NGOs and government institutions introduce lifesaving training to intervene in suicide attempts. For example, swimming would be one of the best lifesaving skills that educational and recreational organizations can offer simply as a sports activity.

## 7. Limitations of the Study and Future Research

The present study contains certain limitations, but we consider these limitations as venues for future investigations. Firstly, the study has been confined to a single district; however, suicide cases happen in other districts of Pakistan also. The findings of this study may have limited external validity. The same study should be replicated to other districts of Pakistan to have a broader view of the phenomenon. Secondly, this study relies on 49 suicide cases, which is apparently a small sample size. Most of the suicide cases are not reported. So, studies in collaboration with police department will help enhance the number of cases for study. Thirdly, the study has used cross-sectional data to predict the causes of suicide. In this respect we suggest longitudinal studies to determine causes with more confidence. Last but not least, the survivals of suicide attempts should be taken for case studies for deeper insights of the phenomenon.

## Figures and Tables

**Table 1:** Summary of the interview themes.

Apparent Causes	Root Causes Explored	Remedies Explored
Domestic Violence	Extended family system Sustainer and depended conflict Mother-in-law and daughter-in-law clashes Inappropriate division of domestic work Possession of domestic and external resources Claim for inherited property by females	Small family units where a single family (parents and children) lives A male should get a separate and independent family setup when he gets married Due right in inherited properties to be divided among all heirs at law Using educational and religious institutions in this regard Police complaint center
Mental Health	Depression Anxiety Retardation Any other permanent disease	Strengthening health organizations and ensuring availability of services of neuro-physicians, psychologists, and psychiatrists Counseling services by NGOs Use of religious and educational institutions.
Marital Complications	Forced marriages Early marriages Out of district and out of clan marriages Polygamy Divorces Failure in love affairs	Legal protection against forced marriages, early marriages, and out of district marriages without having proper information Making permission of prior wife for further marriage mandatory Counseling Police complaint center
Academic Factors	Academic performance College admissions Parental pressure for good grades	Awareness through education Career counseling by teachers Promoting entrepreneurship
Economic Factors	Inability to repay loan Joblessness	Directing the role of micro finance banks Promoting entrepreneurship Use of Zakat for poverty alleviation Proper trial of suicide cases in the court of law Teaching and learning lifesaving skills

**Table 2:** Time period.

Gender	Year			Total
	2017	2018	2019	
Male	3	9	7	19
Female	7	9	14	30
Total	10	18	21	49

**Table 3:** Age.

Gender	Age (in years)							Total
	10–13	14–17	18–21	22–26	27–33	34–40	>41	
Male	2	3	3	3	1	5	2	19
Female	2	6	6	7	3	2	4	30
Total	4	9	9	10	4	7	6	49

**Table 4:** Education.

Gender	Education					Total
	Illiterate	Under Matric	Matric	Intermediate	Bachelor	
Male	3	6	8	2	0	19
Female	14	9	5	1	1	30
Total	17	15	13	3	1	49

**Table 5:** Occupation.

Gender	Occupation					Total
	Jobless	Student	Housewife	Govt. Job	Farmer	
Male	11	4	2	1	1	19
Female	1	6	23	0	0	30
Total	12	10	25	1	1	49

**Table 6:** Marital status.

Gender	Marital Status		Total
	Unmarried	Married	
Male	13	6	19
Female	20	10	30
Total	33	16	49

**Table 7:** Methods of suicide.

Gender	Mode				Total
	Drown	Arm Fire	Poison	Hang	
Male	9	6	2	2	19
Female	21	3	5	1	30
Total	30	9	7	3	49

**Table 8:** Reasons for suicide.

Gender	Mode				Total
	Domestic Issues	Failure in Exams	Insane	Unknown	
Male	6	1	10	2	19
Female	6	3	17	4	30
Total	12	4	27	6	49

## Data Availability

Data will be available on request from the corresponding authors.
